# Genome-Wide Analysis of Long Noncoding RNAs in Porcine Intestine during Weaning Stress

**DOI:** 10.3390/ijms24065343

**Published:** 2023-03-10

**Authors:** Shujie Liu, Xin Tao, Bo Deng, Yongming Li, Ziwei Xu

**Affiliations:** Institute of Animal Husbandry and Veterinary Science, Zhejiang Academy of Agricultural Sciences, 298 Desheng Middle Road, Hangzhou 310021, China

**Keywords:** RNA-seq, piglets, weaning stress, intestine, lncRNA

## Abstract

Long noncoding RNAs (lncRNAs) play crucial roles in various biological processes, and they are considered to be closely associated with the pathogenesis of intestinal diseases. However, the role and expression of lncRNAs in intestinal damage during weaning stress remain unknown. Herein, we investigated the expression profiles of jejunal tissue from weaning piglets at 4 and 7 d after weaning (groups W4 and W7, respectively) and from suckling piglets on the same days (groups S4 and S7, respectively). Genome-wide analysis of lncRNAs was also performed using RNA sequencing technology. A total of 1809 annotated lncRNAs and 1612 novel lncRNAs were obtained from the jejunum of piglets. In W4 vs. S4, a total of 331 lncRNAs showed significant differential expression, and a total of 163 significantly differentially expressed lncRNAs (DElncRNAs) was identified in W7 vs. S7. Biological analysis indicated that DElncRNAs were involved in intestinal diseases, inflammation, and immune functions, and were mainly enriched in the Jak-STAT signaling pathway, inflammatory bowel disease, T cell receptor signaling pathway, B cell receptor signaling pathway and intestinal immune network for IgA production. Moreover, we found that *lnc_000884* and target gene *KLF5* were significantly upregulated in the intestine of weaning piglets. The overexpression of *lnc_000884* also significantly promoted the proliferation and depressed apoptosis of IPEC-J2 cells. This result suggested that *lnc_000884* may contribute to repairing intestinal damage. Our study identified the characterization and expression profile of lncRNAs in the small intestine of weaning piglets and provided new insights into the molecular regulation of intestinal damage during weaning stress.

## 1. Introduction

Early weaning is one of the most stressful events for piglets as they face significant social, environmental, and dietary changes alone. These abrupt challenges seriously impair intestinal mucosa and barrier function, disrupt the intestinal immune system, and increase the susceptibility of piglets to disease and mortality rates [1,2,3]. The early weaning of piglets reportedly results in intestinal villous atrophy and crypt hyperplasia, reduces digestive enzyme activities, and damages intestinal morphology [4,5]. Once the intestinal barrier is disrupted, intestinal permeability increases, which allows luminal bacteria, feed antigens, and toxins to traverse the epithelium and enter systemic tissues. Intestinal inflammation or immunologic responses are further incited, and the production of pro-inflammatory cytokines is enhanced, eventually leading to malabsorption, diarrhea, and systemic disease [6,7]. The worst intestine damage occurs particularly during the first week post-weaning [8,9]. Studies have demonstrated the related signaling pathways and inflammation of weaning stress; however, the molecular mechanism underlying intestinal damage has not yet been clarified.

Long noncoding RNAs (lncRNAs) are defined as a new kind of noncoding RNA (ncRNA) at least 200 nucleotides long. LncRNAs do not encode proteins and are transcribed by RNA polymerase II [10]. Although lncRNAs are initially considered as “transcriptional noise” for long periods, lncRNAs have been found to play critical roles in modulating gene expression at the transcriptional or post-transcriptional levels [11]. Growing evidence has revealed that lncRNAs participate in various biological processes, including disease initiation and development, metabolic and immunological changes, and biotic and abiotic stress responses. They also contribute to the diverse mechanisms as important components [12,13,14,15,16]. Furthermore, lncRNAs usually display low expression levels and poor conservation in different species, take on cell type-, tissue-specific expression [17,18,19]. Recently, many lncRNAs have been identified in humans, mice, pigs, and cattle and are associated with fat deposition, skeletal muscle development, and skin pigmentation [20,21,22]. In addition, the abnormal expression of lncRNAs has been suggested to be involved in the pathogenesis of intestinal diseases including colorectal cancer, Crohn’s disease (CD), and ulcerative colitis (UC) [23,24,25]. Further research on lncRNAs is needed to better understand their potential roles in developmental and pathological states of human or animal intestine.

The intestinal damage of piglets caused by weaning stress may be closely related to the dysregulation of lncRNAs. However, the association between lncRNA and the small intestine of weaning piglets remains unknown. Furthermore, the pig is an important animal model of human diseases, and investigating its intestinal lncRNA transcriptome for disease diagnosis and prevention is necessary. In the present study, we investigated the lncRNA expression profiles in jejunum tissues of weaning piglets and suckling piglets through deep RNA sequencing, and screened intestinal inflammatory- and damage-related candidate lncRNAs. The functions of lncRNA were also evaluated. Our study provided new insights to understand the molecular mechanism of the intestinal damage of piglets during weaning stress and presented potential targets to alleviate intestinal damage.

## 2. Results

### 2.1. Identification of lncRNAs in Piglet Intestine

**Figure 1:**
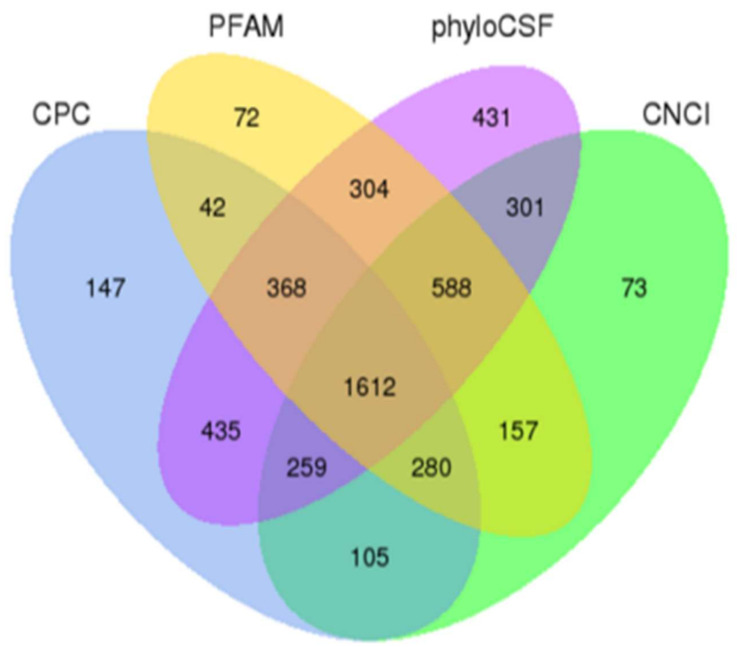
Screening and classification of the candidate lncRNAs in the intestine transcriptome of piglets. Venn diagram illustrating the coding potential analysis by four different tools including CNCI, CPC, Pfam-scan, and phyloCSF.

To identify the intestinal lncRNAs, twelve cDNA libraries from weaning piglets at 4 and 7 d after weaning (groups W4 and W7, respectively) and from sucking piglets on the same days (groups S4 and S7, respectively) were constructed and sequenced with the Illumina HiSeq 2000 platform. On an average of each group, a total of 111,560,697 and 118,340,587 raw reads in W4 and S4, as well as 100,257,893 and 95,055,725 raw reads in W7 and S7 were obtained, respectively. After removing reads with adapter and low-quality reads, the remaining clean reads were obtained, including 108,424,311 and 114,024,937 in W4 and S4, as well as 97,125,151 and 92,236,969 in W7 and S7, respectively. Subsequently, the clean reads about 77.97% and 79.21% in W4 and S4, as well as 78.03% and 76.81% in W7 and S7 were mapped pig reference genome (Susscrofa 10.2) (Supplementary Table S1). In the libraries, transcripts were assembled by Scripture and Cufflinks. After all transcripts from twelve libraries were combined using Cuffmerge, the transcripts with uncertain strand orientation were removed. In total, 150,308 transcripts were obtained. To further minimize the false positive rates in identifying lncRNAs, the transcripts containing a length of ≤200 bp, exons <2, or FPKM <0.5 were discarded. Then, the remaining transcripts were blasted with known reference transcripts, and 1809 known pig lncRNAs as a result were recovered (Supplementary Table S2). Finally, four analytic tools, namely, CNCI, CPC, Pfam-scan, and PhyloCSF, were used to further analyze the coding potential for 5414 transcripts, and a total of 1612 lncRNAs were identified (Figure 1; Supplementary Table S3). Furthermore, the 1612 novel lncRNAs included 1428 lincRNAs (88.6%), 184 anti-sense lncRNAs (11.4%), and 0 intronic lncRNAs (0%). A total of 25,491 protein-coding transcripts were also obtained. In addition, the numbers of annotated lncRNAs were 1747, 1734, 1727 and 1718 in S4, W4, S7 and W7, respectively. The numbers of novel lncRNAs were 1594, 1597, 1593 and 1583 in S4, W4, S7 and W7, respectively. The numbers of mRNAs were 21,416, 21,460, 21,484 and 21,511 in S4, W4, S7 and W7, respectively.

### 2.2. Characteristics of Intestinal lncRNA

The characteristics of piglet intestinal lncRNAs were analyzed. Results showed that the average number of exons of 3421 lncRNAs (1809 annotated lncRNAs and 1612 novel lncRNAs) was 2.57 in the small intestine, and approximately 68.69% and 18.53% lncRNAs contained two exons and three exons, respectively. The average size of the open reading frame (ORF) and length of lncRNAs were 134 and 2809 bp, respectively. We analyzed the differences in length and size of ORF between the two subtypes of lncRNAs. Clear differences in length were found between anti-sense lncRNAs and lincRNAs, with average lengths of 4385 and 2749 bp, respectively. The average sizes of the ORF of the two subtypes of lncRNAs were 146 and 128 bp, respectively (Supplementary Table S2 and Table S3).

**Figure 2:**
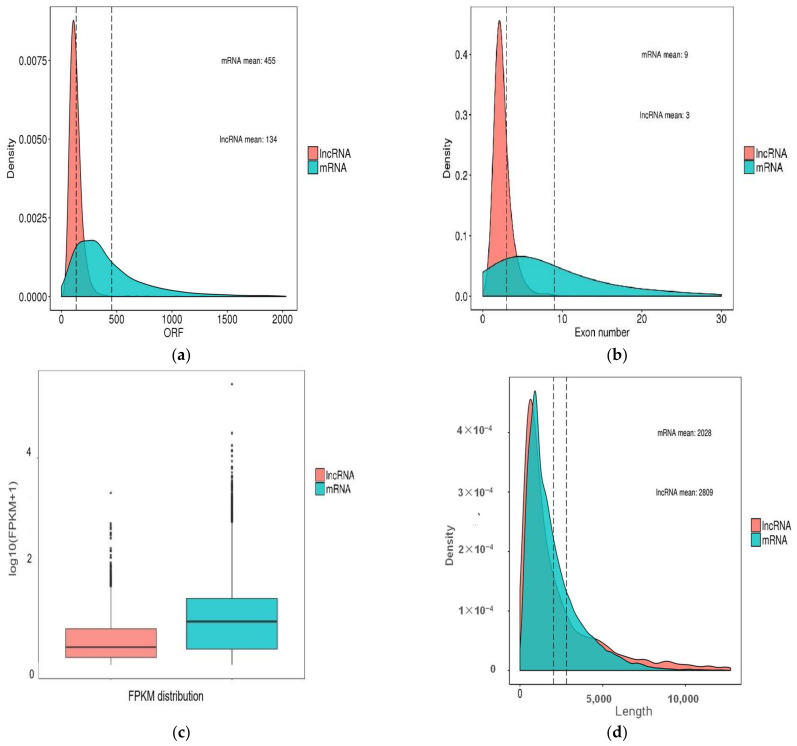
Genomic features of lncRNAs including annotated-lncRNAs and novel-lncRNAs compared to mRNAs. (**a**) ORF length; (**b**) exon number; (**c**) FPKM distribution; (**d**) length.

In order to further show the differences between lncRNAs and mRNAs, comparative analysis of gene feature was performed. Significant differences were observed in the feature between mRNAs and lncRNAs. The ORF sizes of lncRNAs were shorter than those of mRNAs (Figure 2a). The average exon numbers of lncRNAs were fewer than those of mRNAs (Figure 2b). We found that most lncRNAs had a lower expression level with FPKM values than those of mRNAs (Figure 2c). Meanwhile, we found that the average length of lncRNAs was 2809 bp, which was longer than that of mRNA transcripts with 2028 bp (Figure 2d).

### 2.3. Analysis of Differentially Expressed lncRNAs and mRNAs

**Figure 3:**
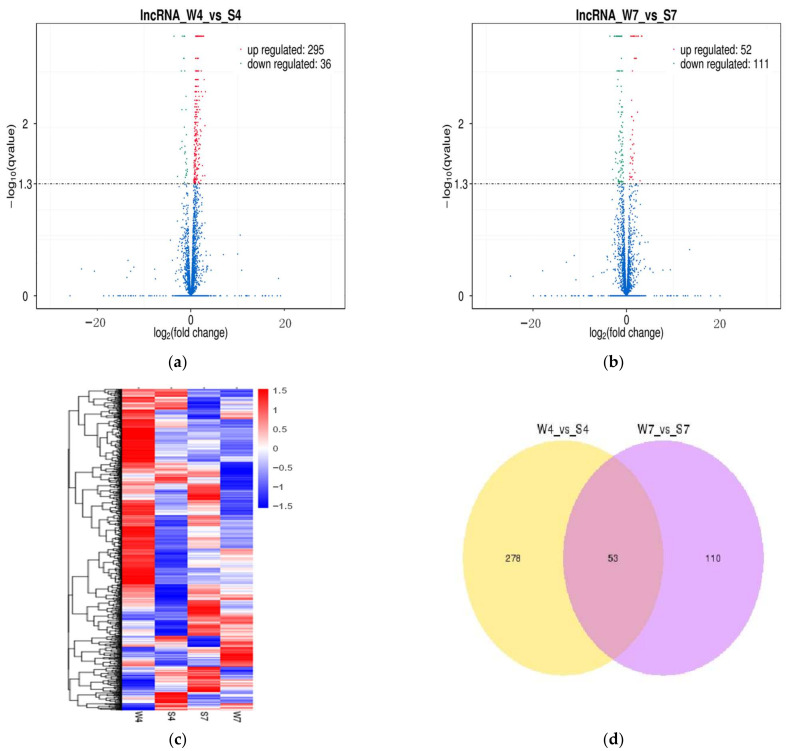
Differential expression analysis of intestinal lncRNAs of piglets between the weaning group and suckling group. (**a**) Volcano plot of DElncRNAs in W4 vs. S4; (**b**) volcano plot of DElncRNAs in W7 vs. S7. (a, b) Red dots represent up-regulated genes, green dots represent down-regulated genes, and blue dots represent non-differentially expressed genes; (**c**) clustered heat map of DElncRNAs based on the FPKM values in the weaning and suckling groups. Red and blue represent high and low expression levels, respectively; (**d**) Venn diagram of common DElncRNAs in W4 vs. S4 and W7 vs. S7.

**Figure 4:**
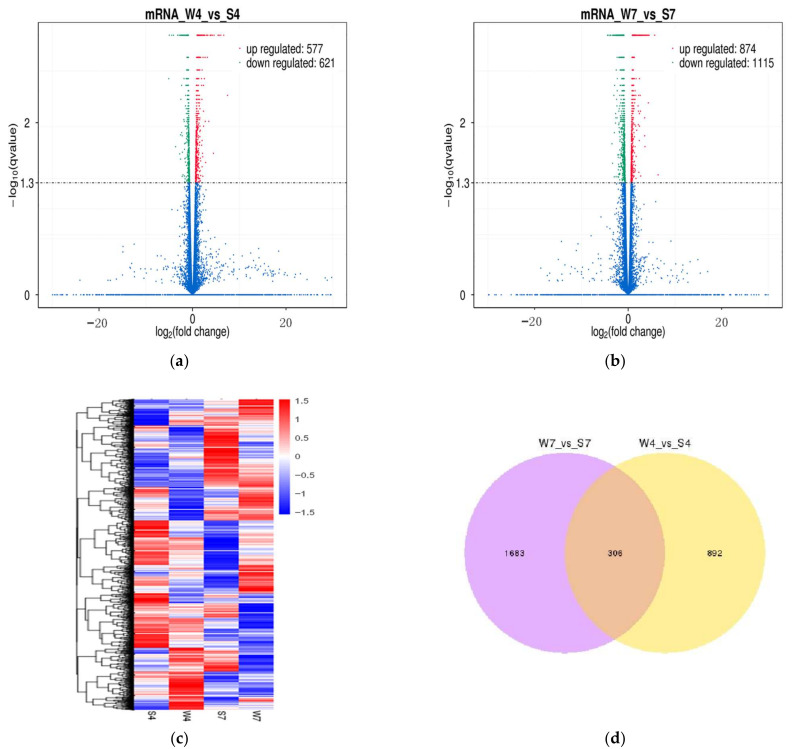
Differential expression analysis of intestinal mRNAs of piglet between the weaning group and sucking group. (**a**) Volcano plot of DEmRNAs in W4 vs. S4; (**b**) volcano plot of DEmRNAs in W7 vs. S7. (**a**,**b**) Red dots represent up-regulated genes, green dots represent down-regulated genes, and blue dots represent non-differentially expressed genes; (**c**) clustered heat map of DEmRNAs based on the FPKM values in the weaning and suckling groups. Red and blue represent high and low expression levels, respectively; (**d**) Venn diagram of common DEmRNAs in W4 vs. S4 and W7 vs. S7.

On the basis of the threshold of FPKM, a total of 3421 lncRNAs were further analyzed to clarify the differential expression of transcripts in the pairwise comparison of intestinal samples (W4 vs. S4; W7 vs. S7). A total of 331 lncRNAs were found to express differently in W4 vs. S4. Among them, 36 were significantly downregulated, and 295 were significantly upregulated in the weaning group (Figure 3a; Supplementary Table S4). Likewise, 163 differentially expressed lncRNAs (DElncRNAs) were identified in W7 vs. S7, including 111 downregulated and 52 upregulated lncRNAs in the weaning group (Figure 3b; Supplementary Table S5). The changing trends of DElncRNAs were similar to a previous study on differentially expressed microRNAs during weaning stress; 98 and 22 differentially expressed microRNAs were identified at 4 and 7 d after weaning, respectively [9]. The heat map also revealed that intestinal lncRNA transcripts are differentially expressed in four groups (Figure 3c). We also found that 53 lncRNAs were common in W4 vs. S4 and W7 vs. S7 (Figure 3d). Similarly, 25491 protein-coding transcripts were analyzed to detect the differentially expressed mRNAs (DEmRNAs) in the pairwise comparison of intestinal samples (W4 vs. S4; W7 vs. S7). In W4 vs. S4, 1198 mRNAs were differentially expressed, among which 577 were upregulated and 621 were downregulated in the weaning group (Figure 4a; Supplementary Table S6). In W7 vs. S7, 1989 DEmRNAs were found in the weaning group, including 874 upregulated and 1115 downregulated mRNAs (Figure 4b; Supplementary Table S7). The heat maps displayed DEmRNAs in four groups (Figure 4c). Additionally, 306 mRNAs were common in W4 vs. S4 and W7 vs. S7 (Figure 4d).

### 2.4. Validation of Differentially Expressed lncRNAs and mRNA

**Figure 5:**
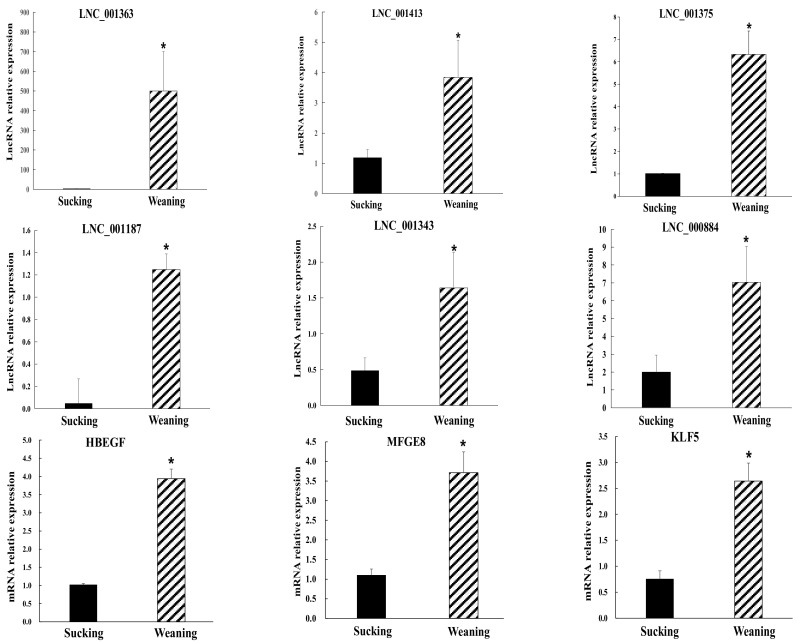
Validation of RNA-seq results by RT-PCR analysis. Relative transcript expression levels were normalized against 18S rRNA using the 2^−ΔΔCt^ method; data are shown as means ± SEM. * *p* < 0.05.

The intestinal injuries of weaning piglets are the most serious at 4 d and somewhat alleviated at 7 d after weaning [9]. Therefore, differentially expressed transcripts were selected in W4 vs. S4 to validate the RNA-seq results. Nine significantly differentially expressed transcripts were selected, including six novel lncRNAs and three mRNAs for quantitative analyses by RT-qPCR (Figure 5). Among the transcripts, *lnc_001343* and *lnc_001363* were randomly selected, and the predicted target genes of other lncRNAs (*lnc_001413*, *lnc_001187*, *lnc_000884*, *lnc_001375*) were primarily involved in intestinal diseases and inflammation response. In addition, the three mRNAs (*HBEGF*, *MFGE8*, and *KLF5*) played important roles in an inflammatory response, regeneration of injured intestine, or stress response. The result of RT-qPCR confirmed that these transcripts were significantly differentially expressed in the intestine at 4 d after weaning, consistent with the RNA-seq findings. Notably, the results of RNA-seq and RT-qPCR showed that nine transcripts were not significantly differentially expressed in W7 vs. S7.

### 2.5. Cis Role of lncRNAs in Target Genes

**Figure 6:**
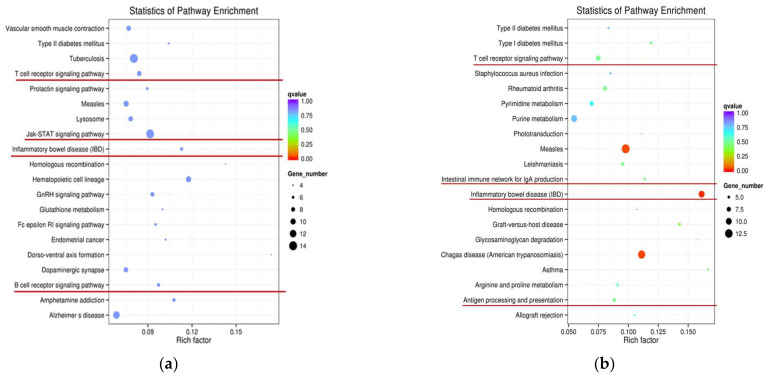
KEGG enrichment analysis of differentially expressed transcripts. axis represented KEGG enriched pathways, and X-axis represented rich factor (rich factor = number of differentially expressed transcripts in this pathway/number of annotated transcripts in background gene set). (**a**) Statistics of pathway enrichment in W4 vs. S4; (**b**) statistics of pathway enrichment in W7 vs. S7.

To evaluate lncRNA function, the potential targets of lncRNAs were further predicted in cis. On the cis action, we searched for protein-coding genes in the regions located 10 or 100 kb upstream and downstream of DE lncRNAs from two comparison groups. Accordingly, in W4 vs. S4, we identified that 119 DElncRNA transcripts corresponded with 179 protein-coding genes within a range of 10 kb, and 269 DElncRNA transcripts corresponded with 888 protein-coding genes within a range of 100 kb. Similarly, in W7 vs. S7, 71 DElncRNA transcripts corresponded with 114 protein-coding genes within a range of 10 kb, and 136 DElncRNA transcripts corresponded with 530 protein-coding genes within a range of 100 kb. GO analysis of cis targets was executed in two comparison groups (W4 vs. S4; W7 vs. S7). Results revealed that three terms were significantly enriched (corrected *p* < 0.05) in W4 vs. S4, and the top ten terms primarily included cellular response to cytokine stimulus, cytokine-mediated signaling pathway, response to cytokine stimulus, CXCR chemokine receptor binding, response to lipopolysaccharide, response to molecule of bacterial origin, and cellular response to organic substance (Supplementary Table S8). However, no significant GO enrichment terms were obtained in W7 vs. S7 (corrected *p* < 0.05), and top ten terms primarily included regulation of dendritic cell differentiation, negative regulation of dendritic cell differentiation, arginine catabolic process, arginine binding and nucleic acid binding transcription factor activity (Supplementary Table S9). Moreover, no significant KEGG enrichment results in W4 vs. S4 (corrected *p* < 0.05), but the top ten terms were primarily involved in various important pathways of intestinal damage and inflammatory response, including the Jak-STAT signaling pathway, inflammatory bowel disease (IBD), T cell receptor signaling pathway, and B cell receptor signaling pathway (Figure 6a; Supplementary Table S10). Furthermore, the pathway of IBD was significantly enriched in W7 vs. S7 (corrected *p* < 0.05). Among the top ten terms, the intestinal immune network for IgA production and T cell receptor signaling pathway were involved in W7 vs. S7 (Figure 6b; Supplementary Table S11). The above-mentioned GO terms and KEGG pathways were all enriched with *p* < 0.05.

### 2.6. Identification of Functional Specificities of lncRNAs

To further understand the relationship between lncRNAs and intestinal damage of weaning piglets, we first selected the pairs of lncRNAs and their target genes, and these pairs of transcripts were significantly differentially expressed between the weaning and suckling groups (corrected *p* < 0.05). Second, these selected pairs were associated with intestinal diseases, inflammation, or immune activation. So, we found that some intestinal lncRNAs acted as specific functional clusters to regulate target genes (Supplementary Table S12). For example, Kruppel-like factor 5 (*KLF5*), an important gene for the maintenance of intestinal crypt architecture and barrier function, was a potential target of *lnc_000884* and *ALDBSSCT0000008940*. Interleukin 21 receptor (*IL21R*) was involved in IBD, cytokine-cytokine receptor interaction, and Jak-STAT signaling pathway, was a potential target of *lnc_001413*, *ALDBSSCT0000011336*, and *ALDBSSCT0000006589*. *CXCL13* is associated with chemokine signaling pathway and cytokine-cytokine receptor interaction, was a potential target of *ALDBSSCT0000010075*, *lnc_001375*, *ALDBSSCT0000011245*, *ALDBSSCT0000008384*, *lnc_001413*, *ALDBSSCT0000011335*, *ALDBSSCT0000011585*, *lnc_000290*, and *lnc_000989*. Moreover, a cluster of lncRNAs targeted the important genes, such as *MFGE8*, *CD22*, *CD19*, *IL4R*, *LTB*, and *CH242-402I11.1* played important roles in IBD, Jak-STAT signaling pathway, T cell receptor signaling pathway, B cell receptor signaling pathway, MAPK signaling pathway, and NF-kappa B signaling pathway. We predicted that these lncRNAs were probably involved in intestinal damage, inflammatory response or homeostasis of weaning piglets. Additional studies are required to verify the definitive function of these lncRNAs.

### 2.7. Lnc_000884 Regulatory Role on Intestinal Epithelial Cell

**Figure 7:**
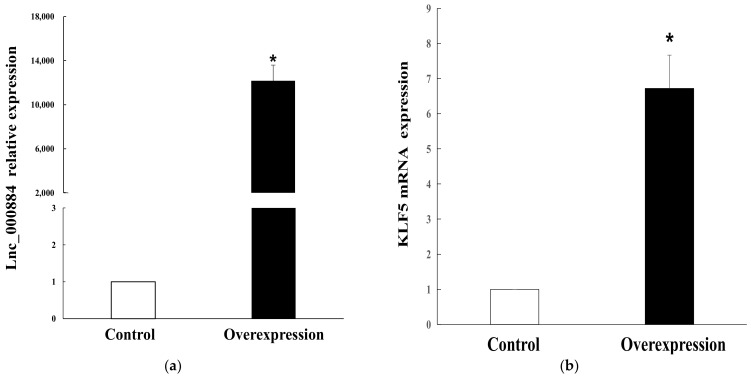
Relative expression of *lnc_000884* and *KLF5* measured by RT-PCR after lnc_000884-transfected into IPEC-J2 cells. The data from three independent experiments were shown as means ± SEM. * *p* < 0.05. (**a**) *Lnc_000884* relative expression; (**b**) *KLF5* relative expression.

**Figure 8:**
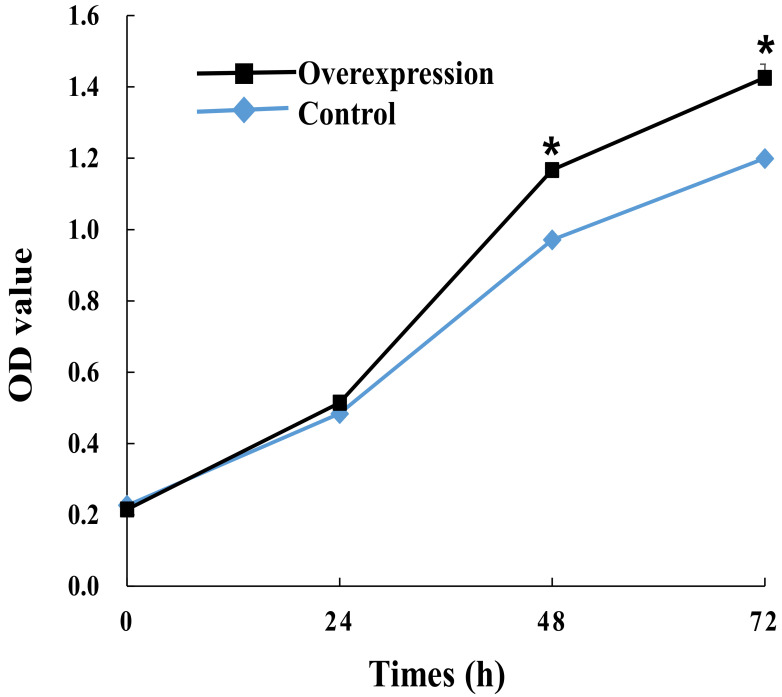
The effects of *lnc_000884* overexpression on IPEC-J2 cell proliferation by MTT assay. The data from three independent experiments was shown as means ± SEM. * *p* < 0.05.

**Figure 9:**
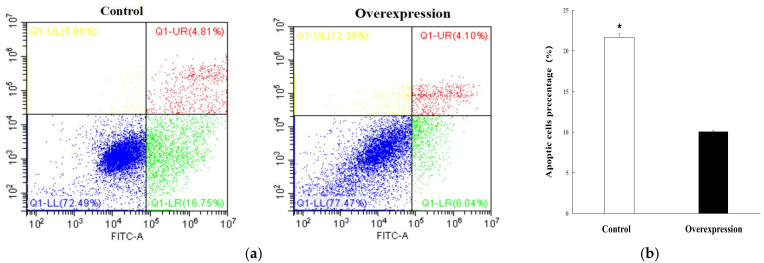
The effects of *lnc_000884* overexpression on IPEC-J2 cell apoptosis. The data from three independent experiments was shown as means ± SEM. * *p* < 0.05. (**a**) The cells’ apoptosis detection by staining with FITC-Annexin; (**b**) the apoptotic cells percentages using a flow cytometer.

To explore the regulatory role of candidate *lnc_000884*, the overexpression vector was constructed and transfected into IPEC-J2 cells. RT-qPCR results showed that *lnc_000884* expression was remarkably enhanced more than 10000-fold in the overexpression group (lnc_000884-transfected cells) than that in the control group (empty-vector-transfected cells) (Figure 7a). Meanwhile, the expression level of *KLF5*, the predicted target gene of *lnc_000884*, was also significantly increased 7.6-fold in the overexpression group compared with that in the control group (Figure 7b). The cell proliferation was assessed by CCK-8 at 24, 48, 72 h after transfection. OD value is the absorbance at 450 nm, and indirectly reflects the number of living cells (Figure 8). Compared with the control group, IPEC-J2 cell proliferation was significantly enhanced in the overexpression group at 48 h, and 72 h after transfection. CCK-8 assay results showed that *lnc_000884* overexpression significantly increased the proliferation rates of IPEC-J2 cell. Finally, the effect of *lnc_000884* on IPEC-J2 cell survival was determined with an Annexin V-FITC apoptosis detection kit. The percentages of early and late apoptotic cells were approximately 16.75% and 4.81% in the control group, 6.04% and 4.10% in the overexpression group, respectively (Figure 9a). The percentage of total apoptotic cells in the control group was significantly higher than that in the control group (Figure 9b). Results displayed that *lnc_000884* overexpression inhibited cell apoptosis.

## 3. Discussion

LncRNAs reportedly play important roles in various biological processes, but little is known about the interactions between lncRNAs and intestinal diseases. In the present study, intestinal tissues from weaning and suckling piglets were selected to investigate DElncRNAs and explore their functions. We found that the basic characteristics and types of intestinal lncRNAs in piglets were similar to those in other mammalian species [21,22]. Compared with protein-coding transcripts, identified lncRNAs were shorter in ORF size, and had fewer exon numbers and lower expression levels. However, the lncRNAs were longer on average than the protein-coding transcripts. We also found that the lncRNAs in the piglet intestine (2809 bp) were longer than those in pig skeletal muscle (1043 bp), endometrium (1454 bp), and testicular tissues (1240 bp) [26,27,28]. Additionally, the total numbers of lncRNAs in piglet intestine (3421) were considerably higher than those in pigs’ skeletal muscle (570) and testicular tissues (777), but were lower than those in the endometrium (4953) [26,27,28]. These findings indicated that our newly identified lncRNA appeared to be highly intestinal-type specific, and suggested that lncRNAs participated in specific biological regulation with their diverse functions.

LncRNAs contribute to their functions to diverse stressful stimuli [29]. In the present study, we screened out DElncRNAs in the small intestine of piglets after weaning, and the number of DElncRNAs was about two-fold at 4 d than that at 7 d after weaning. This indicated that weaning stress caused dysregulated expression of intestinal lncRNAs, and these DElncRNAs may play critical roles in the process of intestinal damage with the phase-specific function. Furthermore, the number of DElncRNA was quite possibly related to the severity of intestine damage. Similar to our results, studies reported that intestine disadvantages of piglets deteriorated at 4 d after weaning and then gradually relieved. The number of differentially expressed microRNAs of piglets’ intestine was also significantly increased at 4 d than that at 7 d after weaning [9]. In ileum and rumen tissues of calves, a total of 14 and 525 lncRNAs were significantly differentially expressed between D33 (pre-weaning) and D96 (post-weaning), respectively [30]. In addition, several studies have reported that lncRNAs participated in intestinal stress or disease processes. CD patients reportedly have eight DElncRNAs (four up- and four downregulated) in pathological mucosa from the ileal end [31]. Radiation-induced intestinal damage reveals that 91 lncRNAs are significantly upregulated and 57 lncRNAs are downregulated in murine jejunum [32]. Infection with porcine endemic diarrhea virus induces lncRNA differential expression in piglet ileum; lncRNA_9606 is significantly upregulated 300-fold [33]. All these results demonstrate the regulatory roles of lncRNA in intestine-related diseases.

Inferring the potential function of lncRNAs is vital to elucidate their roles in the process of intestinal injuries caused by weaning stress. Consequently, we predicted the functions of DElncRNAs by searching cis target genes. We found that many lncRNAs were mostly involved in inflammatory response or immune functions through the upregulation of target genes, which may serve essential roles in the development of intestinal damage. For example, *lnc_001413*, *ALDBSSCT0000011335*, *ALDBSSCT0000011585*, and *lnc_000785*, etc. were highly upregulated in the intestine of weaning piglets and found to target *CD19*. CD19 is expressed on the B cell surface as a co-receptor and is involved in the B cell receptor signaling pathway, primary immunodeficiency, and PI3K-Akt signaling pathway [34]. *ALDBSSCT0000010075*, *lnc_001375*, and *ALDBSSCT0000011245*, etc. target the gene *CXCL13*, which is known as B cell-attracting chemokine 1 or B-lymphocyte chemoattractant, regulates the homing of B cells and subsets of T cells to lymphoid follicles by binding to receptor CXCR5 [35]. Furthermore, these lncRNAs also display some important activities in inflammatory diseases. *ALDBSSCT0000006617*, *lnc_001187*, *lnc_000881*, and *lnc_001413* target *CD22*, which is expressed in mature B cell lineages and contributes to the sensitive control of B-cell response to antigen as a B cell co-receptor [36]. *Lnc_001413*, *ALDBSSCT0000011336*, *ALDBSSCT0000006589*, and *ALDBSSCT0000006589* have been found to target *IL21R*, which is significantly highly expressed in the intestine epithelium of patients from CD and UC, and the IL-21/IL-21R signaling may play an important role in the pathogenesis of IBD [37]. *ALDBSSCT0000006589* targets the IL-4 receptor (*IL-4R*), and IL-4 transmits signals into the cellular nucleus and acts its functions in the process of inflammation [38,39]. Accumulating evidence has revealed that intestinal inflammation plays a critical role in driving the impairment of the intestinal barrier at early weaning of piglets [3,40]. In the present study, many lncRNAs targeted inflammatory cytokines with significant upregulation of their expression, these indicate that lncRNAs’ regulatory reactions may be one of the core features of the pathogenesis of intestinal damage in weaning piglets.

Furthermore, GO functional annotation was conducted for DElncRNAs and protein-coding genes. Results showed that high enrichment was involved in the cellular response to cytokine stimulus, cytokine-mediated signaling pathway, response to cytokine stimulus, CXCR chemokine receptor binding, response to lipopolysaccharide, response to molecule of bacterial origin, and cellular response to organic substance. KEGG pathway analysis further showed that all enriched pathways were involved in IBD, Jak-STAT signaling pathway, T cell receptor signaling pathway, B cell receptor signaling pathway, intestinal immune network for IgA production, and antigen processing and presentation, which were closely associated with weaning-induced inflammation and immunization. These regulatory signaling pathways play important roles in intestinal damage. For example, IBD is characterized by the relapsing inflammation of the gastrointestinal tract because of unbalanced activation of the mucosal immune system in response to luminal antigens [41]. The Jak-STAT signaling pathway is implicated in the pathogenesis of inflammatory and autoimmune diseases including intestinal diseases, and is a key signaling mechanism for growth factors and cytokines [42,43]. In particular, JAK inhibitors have been shown to affect several pro-inflammatory cytokine-dependent pathways [44]. The intestinal immune network for IgA production, and antigen processing and presentation are involved in disturbances in the intestinal barrier of weaning piglets, resulting in the translocation of luminal bacteria, antigens, and toxins to the inner layers of tissues and inciting systemic inflammatory and mucosal responses [45]. At 4 d after weaning, KEGG pathways of microRNA target genes in piglets’ intestines mainly included cytokine-cytokine receptor interaction, cell adhesion molecules (CAMs), chemokine signaling pathway, antigen processing and presentation and T cell receptor signaling pathway, and so on [9]. In a colitis mouse model, the core differentially expressed mRNAs were markedly enriched in the cytokine-cytokine receptor interaction and chemokine signaling pathways [46]. These results further reveal the main participation of intestinal lncRNAs in the intestinal inflammatory response and immune functions.

Among the differentially expressed lncRNA-associated mRNAs, we found that lncRNAs and their target genes were also involved in intestinal cell proliferation, differentiation, or apoptosis. This involvement may be important for the intestinal repair of weaning piglets by regulating their expression following intestinal inflammation and damage. In the present study, *lnc_000884* and the target gene *KLF5*, as well as *lnc_001413* and the target gene *MFGE8*, were significantly upregulated at 4 d after weaning. MFGE8, a glycoprotein secreted by various cells, is involved in the phagocytic clearance of apoptotic cells, effectively attenuating inflammation, promoting intestinal mucosal healing, and maintaining mucosal integrity in DSS and TNBS-induced mouse colitis [47,48]. MFGE8 production is reportedly downregulated during the initiation of colitis, whereas it increases from the healing phase to restore homeostasis mechanisms [49]. KLF5, a zinc-finger transcription factor, is expressed in intestinal epithelium and is crucial to maintaining the normal barrier function and crypt architecture of intestinal epithelia [50]. KLF5 has also been shown to promote intestinal epithelial cell proliferation, differentiation, and apoptosis [51]. In colonic tissues, constitutive KLF5 overexpression protects against murine colitis under the inflammatory stimulus [52]. In the present study, the overexpression of *MFGE8* and *KLF5* may be important for piglets to initiate healing and further promote the repair of the damaged intestines after weaning. To further explore lncRNA roles in the intestine, we selected *lnc_000884* and constructed its overexpression vector. Transfection with pLVX-IRES-lnc_000884-puro successfully resulted in the overexpression of *lnc_000884* by more than 10,000-fold, and the production of the target gene *KLF5* was increased by 7.6-fold in IPEC-J2 cells. Additionally, *lnc_000884* overexpression significantly promoted the proliferation and depressed apoptosis of IPEC-J2 cells. These findings suggested an essential role of *lnc_000884* in the process of intestinal damage and repair. Increasing evidence has revealed that KLF5 expression is activated by various external stimuli including various bacterial pathogens or intestinal damage, and acts as a key regulator of stress responses [53,54]. However, it was still unclear whether *KLF5* was a major gene affected by *lnc_000884* in this study, and more studies are needed to explore the accurate regulatory mechanism of *lnc_000884* in the process of intestinal damage and repair.

In the present study, we identified novel lncRNAs in the porcine intestine, which greatly increased the number of porcine lncRNAs. Our study showed the lncRNA and mRNA expression profiles from weaning and suckling piglets during weaning stress. Bioinformatic analysis suggested that some lncRNAs were involved in important biological processes associated with intestinal damage, inflammation, and immune functions during weaning stress, and may play important roles in intestinal diseases. In particular, *lnc_000884* overexpression promoted the proliferation, and inhibited the apoptosis of IPEC-J2 cells, which may contribute to repairing the intestine damage of weaning piglets. Our study provided new insights into the molecular mechanism of intestinal damage during weaning stress, and also contributed to further research in intestinal diseases of humans and other animals.

## 4. Materials and Methods

### 4.1. Experimental Animals and Sample Preparation

Two newborn litters of Duroc piglets, a total of twelve piglets (six per litter), with the same age and similar genetic background were selected from the Haining Technology Farm of Zhejiang Academy of Agricultural Sciences (Hangzhou, China). Their sows with gilts were housed in pens with farrowing crates. Six piglets (three per litter) were randomly selected and separated from sows at 21 days of age. These weaning piglets were raised in another pen and offered ad libitum access to water and a starter diet. The other six piglets (three per litter) remained with the sows and were allowed to continue suckling.

Each three from the weaning piglets (n = 6) were humanely euthanized after intraarterial injection of pentobarbital at 4 (n = 3) and 7 d (n = 3) after weaning (groups W4 and W7, respectively). In the same way, each three piglets from suckling piglets (n = 6) were humanely euthanized at 25 (n = 3) and 27 d (n = 3) of age (groups S4 and S7, respectively). The abdominal cavity of piglets was opened along the midline, and approximately 2 cm sections of intestinal tissue were sampled from distinct regions of the distal jejunum (65 cm prior to the jejunoileal junction) [26]. Twelve intestinal samples were rapidly frozen in liquid nitrogen and stored at −80 °C for RNA extraction

### 4.2. RNA Isolation and Qualification

Total RNA was isolated from the intestinal sample of each individual by using TRIzol reagent (Invitrogen, Carlsbad, CA, USA) following the manufacturer’s instruction. RNA contamination and degradation were monitored with 1% agarose gel. RNA purity and concentration were measured with a NanoPhotometer spectrophotometer (IMPLEN, Westlake Village, CA, USA) and Qubit RNA Assay Kit in Qubit 2.0 Fluorometer (Life Technologies, Carlsbad, CA, USA), respectively. RNA integrity was assessed using RNA Nano 6000 Assay Kit by Bioanalyzer 2100 system (Agilent Technologies, Santa Clara, CA, USA).

### 4.3. Library Preparation for lncRNA Sequencing

For RNA sample preparations, 3 μg of RNA from the jejunum of each piglet served as the input material. First, ribosomal RNA was removed using Epicentre Ribo-zero™ rRNA Removal Kit (Epicentre, Madison, WI, USA), and then rRNA-free residue was cleaned by ethanol precipitation. Sequencing libraries were then generated using rRNA-depleted RNA by NEBNext^®^ Ultra™ Directional RNA Library Prep Kit for Illumina^®^ (NEB, Ipswich, MA, USA) according to the manufacturer’s recommendation. First- and second-strand cDNAs were synthesized, and the remaining overhangs were subsequently turned into blunt ends by exonuclease/polymerase activities. After adenylation of the 3′ ends of DNA fragments, NEBNext Adaptor with a hairpin loop structure was ligated to prepare for hybridization. The library fragments were purified to select cDNA fragments preferentially 150–200 bp in length. Then, USER Enzyme (NEB, Ipswich, MA, USA) was used with the size selected and adaptor-ligated cDNA at 37 °C for 15 min followed by 5 min at 95 °C before PCR. Furthermore, PCR was performed with Phusion High-Fidelity DNA polymerase, Universal PCR primers, and Index (X) Primer. Finally, the products were purified (AMPure XP system) and library quality was assessed with Agilent Bioanalyzer 2100 system. After cluster generation, twelve independent libraries were sequenced on an Illumina Hiseq 2500 platform at the Novogene Bioinformatics Institute (Beijing, China), and 100 bp paired-end reads were generated.

### 4.4. Quality Control and Transcriptome Assembly

Raw data (raw reads) were first processed through in-house Perl scripts, and clean data (clean reads) were obtained by removing reads containing adapter, reads containing ploy-N, and low-quality reads. At the same time, the Q20, Q30, and GC content of the clean data were calculated. All downstream analyses were based on the clean data with high quality. Paired-end clean reads were aligned to the reference genome (Scrofa 10.2) by using TopHat v2.0.9. Finally, the mapped reads of each sample were assembled by Scripture (beta2) and Cufflinks (v2.1.1) in a reference-based approach. The scripture was run with default parameters, Cufflinks was run with ‘min-frags-per-transfrag = 0’ and ‘library-type’, other parameters were set as default.

### 4.5. Coding Potential and Expression Analysis of Transcriptome

Four analytical tools including CNCI, CPC, Pfam-scan, and PhyloCSF were used to identify candidate lncRNAs. We filtered out the transcripts that were predicted with coding potential by any or all of the four tools above, and those without coding potential were deemed as the candidate set of lncRNAs. Quantification of gene expression level was assessed by calculating the FPKMs of the transcripts with Cuffdiff, and gene FPKMs were computed by summing the FPKMs of the transcripts of each gene group. Cuffdiff provided statistical routines to determine the differential expression in digital transcript or gene-expression data by using a model based on the negative binomial distribution. Transcripts or genes with an adjusted *p*-value < 0.05 were assigned as differentially expressed.

### 4.6. Target Gene Prediction and Functional Enrichment Analysis

The target genes of lncRNAs in cis were predicted to explore the function of lncRNAs. Coding genes 10 or 100 kb upstream and downstream of lncRNAs were searched, and then their function was analyzed. The GOseq R package was used to implement Gene Ontology (GO) enrichment analysis. KOBAS software was used to test the statistical enrichment of target genes in KEGG pathways. Additionally, the GO terms and KEGG pathways with a corrected *p* value <0.05 were considered significantly enriched.

### 4.7. Quantitative Real-Time RT-PCR

RNA samples of 12 piglets used for the RNA-seq experiment were analyzed by quantitative real-time PCR (RT-qPCR). cDNA synthesis was performed using ReverTra Ace reverse transcriptase (Toyobo Co., Osaka, Japan) following the manufacturer’s instruction. RT-qPCR was performed using a StepOne Plus real-time PCR system with SYBR Green master mix (Applied Biosystems, Singapore). Specific primers were designed according to the sequences of selected transcripts using the NCBI (Supplemental Table S1). Each real-time RT-PCR reaction in 20 μL of reaction mixture comprised 10 μL of KOD SYBR^®^ qPCR Mix (TaKaRa, Dalian, China), 0.4 μL of 50 × ROX reference dye, 0.2 μL of each primer, 2 μL of cDNA, and 7.2 μL of RNase-free dH_2_O. Swine 18S rRNA was used as an internal control gene, and all samples were analyzed in triplicate. The relative expression levels of genes were determined using 2^−ΔΔCt^ value methods. All data were analyzed by Student’s *t*-test using SPSS version 21.0 statistical software. RT-qPCR data were presented as the mean ± standard deviation, and *p* < 0.05 was considered statistically significant.

### 4.8. Construction of lnc_000884 Overexpression Vector and Transfection of Cells

From differentially expressed transcripts in W4 vs. S4, we selected *lnc_000884* to analyze its regulating function on intestinal epithelium cells. IPEC-J2 cells from the porcine jejunal cell line originate from the mid-jejunum of neonatal piglets and share similar characteristics with intestinal epithelium. To construct the *lnc_000884* overexpression vector in IPEC-J2 cell, the full length of *lnc_000884* cDNA was synthesized in GeneCreate Biological Engineering Co., Ltd. (Wuhan, China) and then subcloned into the pLVX-IRES-Puro vector (plnc_000884). For transfection, IPEC-J2 cells were cultured in DMEM/F12 /medium (Gibco, Carlsbad, CA, USA) supplemented with 10% fetal bovine serum (Gibco, Carlsbad, CA, USA), 1% penicillin–streptomycin (Sigma, St. Louis, MO, USA), and 1% glutamine (Sigma, St. Louis, MO, USA). Furthermore, IPEC-J2 cells were cultured up to 30% confluency and transfected with plnc_000884 (as the overexpression group) or empty vector (as the control group) by replacing the transfection medium. Cell samples were collected for subsequent analyses 48 h after the transfection. The relative expression levels of *lnc_000884* and predicted target genes, *KLF5*, in transfected cells were examined using RT-qPCR.

### 4.9. Cell Proliferation and Apoptosis Detection

CCK-8 assay was performed to assess the effect of *lnc_000884* on IPEC-J2 cell viability. Transfected cells were seeded onto 96-well plates at a density of 1000 cells/well and then cultured for 24, 48, and 72 h. After incubation with 10 μL of CCK-8 (Dojindo, Kumamoto, Japan) at 37 °C for 2 h, the number of viable cells was evaluated by measuring optical density at 450 nm using a Multiskan MK3 microplate reader (Thermo, Waltham, MA, USA).

Cell apoptosis was measured using an AnnexinV-FITC Apoptosis Detection kit (Beyotime Institute of Biotechnology, Shanghai, China). After transfection and culturing for 48 h, the cells were washed twice with cold phosphate-buffered saline and then resuspended in the binding buffer containing Annexin V/propidium iodide. Cells were then incubated for 15 min in darkness, and apoptosis was analyzed by flow cytometry.

## Data Availability

Data are contained within the article or Supplementary Materials.

## Supplementary Materials

The following supporting information can be downloaded at: https://www.mdpi.com/article/10.3390/ijms24065343/s1.
